# Enhanced QoS Routing Protocol for an Unmanned Ground Vehicle, Based on the ACO Approach

**DOI:** 10.3390/s23031431

**Published:** 2023-01-28

**Authors:** Ali M. Ali, Md Asri Ngadi, Rohana Sham, Israa Ibraheem Al_Barazanchi

**Affiliations:** 1 Department of Computer Science, Faculty of Computing, University Technology Malaysia, Johor Bahru 81310, Malaysia; 2 School of Business, Asia Pacific University of Technology, and Innovation, Jalan Innovasi 6, Technology Park Malaysia, Kuala Lumpur 57000, Malaysia; 3 Computer Engineering Techniques Department, Baghdad College of Economic Sciences University, Baghdad 10, Iraq; 4 College of Engineering, University of Warith Al-Anbiyaa, Karbala 56001, Iraq

**Keywords:** ACO, QoS, VANET, firefighting UGV, V2X, RS

## Abstract

Improving models for managing the networks of firefighting unmanned ground vehicles in crowded areas, as a recommendation system (RS), represented a difficult challenge. This challenge comes from the peculiarities of these types of networks. These networks are distinguished by the network coverage area size, frequent network connection failures, and quick network structure changes. The research aims to improve the communication network of self-driving firefighting unmanned ground vehicles by determining the best routing track to the desired fire area. The suggested new model intends to improve the RS regarding the optimum tracking route for firefighting unmanned ground vehicles by employing the ant colony optimization technique. This optimization method represents one of the swarm theories utilized in vehicles ad–hoc networks and social networks. According to the results, the proposed model can enhance the navigation of self-driving firefighting unmanned ground vehicles towards the fire region, allowing firefighting unmanned ground vehicles to take the shortest routes possible, while avoiding closed roads and traffic accidents. This study aids in the control and management of ad–hoc vehicle networks, vehicles of everything, and the internet of things.

## 1. Introduction

Vehicle ad–hoc networks (VANETs) are growing in popularity in the industry and scientific communities as an essential component of smart cities and modern societies [1]. In general, there are three ways of wireless communication in this type of network, vehicle-to-vehicle (V2V) connection, vehicle-to-infrastructure (V2I) connection, and vehicle-to-everything (V2X) connection [2]. VANET applications include collision warning applications and driver assistance, classified as public safety and driver information applications. These applications employ public services, including service vehicles in developed cities, such as driving driverless fire vehicles to the fire spot in the quickest and shortest routes possible, as well as avoiding road obstructions and congestion [3].

VANETs, with their mobility and self-regulation across area networks, are the appropriate solution for managing firefighting unmanned ground vehicles (firefighting UGV). However, many issues remain in administering these networks, including the scalability, routing, privacy, security, and quality of service (QoS) [4]. The primary goal of this research is to analyze the QoS problem and the network routing problem with high efficiency, to obtain a better stability in the routing protocols of large-scale dynamic VANET networks, where VANET routing protocols still suffer from various restrictions [5].

Some research has employed VADD and A-STAR to address the problem of QoS, but it remains a theory that is not appropriate for dealing with dynamic networks, such as VANETs, but rather for structured networks [6]. Other research employed SADV to redirect packets and increase the QoS in real time. However, this strategy might cause network congestion, especially during peak hours, which creates a bottleneck problem in emergency scenarios [7]. Moreover, some researchers have employed local motion characteristics, such as the GyTAR and TADS protocols, to predict the next junction, although neither the density of the chemicals nor their distribution can reflect the overall pattern of the QoS improvement. The increased vehicle density can improve the network connectivity, but it also can cause an increase in the data throughput on the network [4]. The CAR protocol for the routing packets employs the idea of blind flooding in its schemes, rendering it ineffectual in improving the QoS and potentially burdening the network [8].

According to prior research, the primary issues that VANETs have in constructing acceptable networks for services are: the path inaccuracy, the ineffectiveness of the QoS assessment in the traditional or incomplete protocols, and their inability to adapt to the changes in the network architecture. Meeting the requirements for the implementation speed and efficiency has become an essential demand in modern cultures. For a more in-depth evaluation of prior studies, along with their limitations, has been shown in Table 1. In this research, the network throughput is proposed as a factor to measure the fastest route for firefighting UGVs to reach the fire region because it employs an unstructured network of VANETs to communicate among the firefighting UGVs, that are represented by a group of wireless mobile nodes, each node representing a firefighting UGV. The ant colony optimization (ACO) theory is utilized to tackle the problem of selecting the best path and avoid accidents and collisions. To choose the optimal route, the firefighting UGVs apply the principle of opportunism, rather than the theory of deep flooding. Following the selection of the best tracking route for the firefighting UGV, the VANETs use the selected road to direct all firefighting UGVs and internally increase the QoS.

The following are the primary contributions of this paper:

1. Contribute to the presentation of a model diagram depicting how firefighting UGVs will interact with one another in the case of a fire, discuss the optimization of the QoS based on the throughput factor and select the fastest access method;
2. Utilize the ACO approach to find the best tracking route between the fire station and the fire area, to achieve the best QoS;
3. Create a mathematical model to demonstrate the magnitude of the QoS improvement obtained after implementing the proposed approach.

The rest of the article is structured as below:

The next section proposes the methodology of the suggested technique. The third and fourth sections describe the results and discussion, then the article ends with the conclusion.

## 2. Method

**Figure 1:**
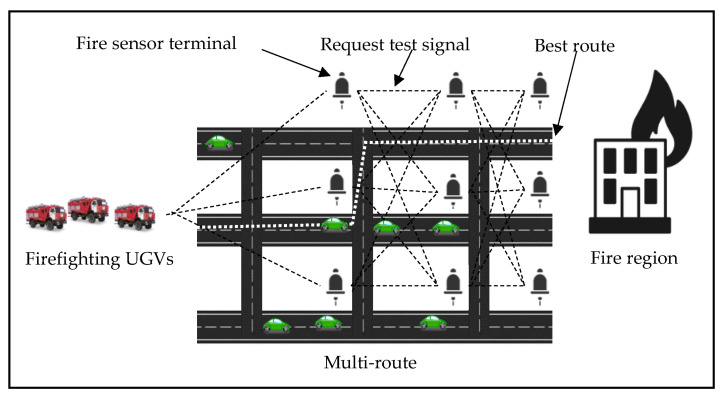
ACO scenario of the VANET for the firefighting UGVs, which depends on the throughput parameter to select the optimal route.

As illustrated in Figure 1, we discuss the VANET protocol that is boosted by the ACO in this section. VANETs are adaptive intersection-based routing systems that employ the suggested method to find the suitable tracking path of the QoS. Through leveraging the node throughput, the QoS is quantified from the side of the connection likelihood, the ratio of the packet delivery, and the latency. Prior to sending data packets to the shortest path, each firefighting UGV sends a request for routing to the nearby fire sensor terminal and receives throughput for the available routing information. If such routing information exists, an affirmative answer is delivered back to the firefighting UGV, which instantly commences the data packet forwarding and subsequently directs all firefighting UGVs into the fire region through the designated route. Otherwise, the fire sensor terminal transmits a negative message to the firefighting UGV, and subsequently, depending on the ACO protocol, all firefighting UGVs quit this route. The novel ACO for AVNETs is made up of essentially two components; the optimal route establishment and the terminal intersection selection.

The ACO’s advantages make it appropriate for the dynamic applications where positive feedback leads to the rapid identification of good solutions. It might also be a distributed computation, which avoids the premature convergence.

### 2.1. ACO-Based Optimization

The ant colony optimization method, utilized to solve multi-object problems with a high computing complexity, was inspired by the food-foraging activities of actual ants [9,10,11].

The ACO may also be appropriate for the VANET routing issues for the following reasons [12,13]:

1. The number of ACO nodes, namely scalability, vary depending on the size of networks, which is enhanced by the interactions between various ant colonies. The ant can be portrayed as a firefighting UGV.
2. Adaptation: As the network evolves, the number of ant colonies can be increased or decreased.
3. The strategy of the parallelism for the ant colony: this technique aids in the acceleration of the processes.
4. Fault tolerance: Because the ACO is an uncentralized control mechanism, several connections that are lost will have no effect on the overall system.

Depending on the previous considerations, the ACO flexibility and the nature of decentralization, together are essential to address the obstacles of tracking the routing optimization. The ACO is employed in the development of a multi-objective heuristic algorithm that determines the optimum path, based on the shortest distance and the lowest chance of disconnection [14]. It is a hybrid routing system that combines the reactive route construction with the proactive route management. Nonetheless, each node (firefighting UGV) is required to keep all traffic from the visited routes, which observes the network of remote routers [15].

### 2.2. V2X-Based Optimization

The V2X routing protocol for the VANETs was intensively researched [16,17,18]. The V2X routing protocol predicts a series of valid intersections to transfer the data packets from the firefighting UGVs to each other and the fixed infrastructures in the urban situations [19,20,21]. These links are calculated using the geographic locations, the speed, acceleration, and the vector direction of the UGV. The V2X, moreover, disregards the real-time traffic data (such as vehicle density) inside the road segments. The ACO is suggested to enhance the behavior of the UGVs in the environments of the V2X depends on the vehicle motion specifications and the intersections among vehicles and the units in the side road [22], in this study, the ACO algorithm scheme’s algorithmic information is adjusted to accomplish a greater rate of closure and stable VANET [23]. An interested low latency protocol is called “DUBHE” and is used to transport information of the firefighting UGVs to the infrastructure. The protocol consists from the delay, path, and broadcast parameters [24]. The IGRP is a V2X routing system, based on the efficient road intersection selections, in terms of the network connectivity, latency, error rate, and capacity [25]. The IGRP, moreover, requires a full route and is incapable of dealing with fast topology changes in the systems of the VANET. In the VANET, an adaptation technique, based on the throughput of the ACO route is suggested to achieve an improved efficiency. To begin, we develop an ACO-based technique for calculating the best tracking route. Furthermore, Instead than following the whole path provided in the packet headers, the data packets are transmitted per intersection, as is the case with other source-driven routing protocols, to boost the routing stability, respond to topology changes, and minimize the network cost. Furthermore, we developed the theoretical criteria to predict the QoS of road segments, in terms of the connection likelihood, latency, and delivering ratio.

### 2.3. The Suggested Scheme

**Figure 2:**
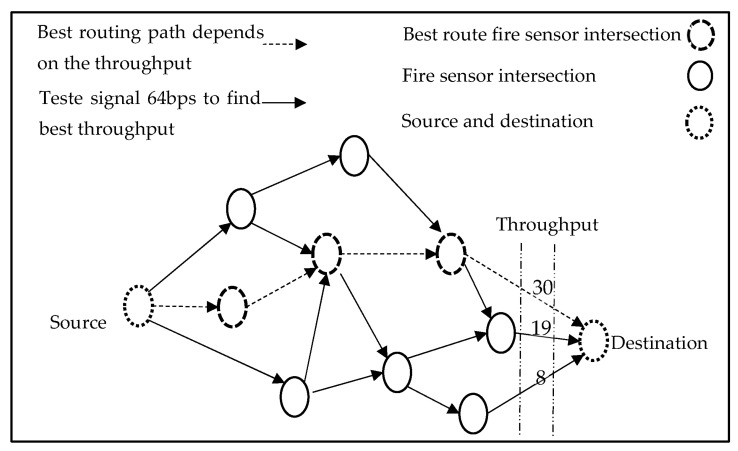
Neural network procedure for the VANET protocol boosted with the ACO algorithm.

In this study, each firefighting UGV is supplied with a digital map, GPS, and navigation model that gives information to the vehicle, such as the network throughput, speed, direction vector, locations, fire sensor intersections, and road segment length. Furthermore, the firefighting UGVs would use location services to establish their destinations’ geographic locations, and various communication pairs would have the same QoS qualification. Moreover, at each junction, a static node (SN) is deployed as a fire sensor, to aid the in packet forwarding and routing information storage. The VANET with the ACO seeks to discover the optimum route connection, the throughput, and latency, instead of fulfilling the time limitation in the urban settings. $\mathrm{The}\;D\;\left(I,E\right)$ graph represents the street map with a fire sensor connecting any two nearby junctions. We can clearly describe the best tracking route (y) as a row of intersections (I1, I2, …, In1, In), which are connected by a set of fire sensors (c1, c2, …, cm), where n=n1. It is worth noting that “I1” is the first junction linked to the firefighting UGV F, and “In” is the last intersection linked to the destination. In urban situations, the VANET supported with the ACO, aims to achieve the optimal route, the QoS, the throughput, and the latency delay limit. The fire sensor connects any two adjacent intersections given in the $D\;\left(I,E\right)$ urban street map. We can clearly describe the best backbone route y as a series of junctions I1, I2, …, In, which are connected by a set of fire sensors e1, e2, …, em, where m=n−1. It is worth noting that “I1” is the first junction linked to the source firefighting UGV and “In” represents the last intersection linked to the firefighting UGV destination. “I1” has been assigned to the source terminal (ST) and “Im” to the destination terminal (IM) (DT). Based on the foregoing, the VANET routing problem may be stated as an optimization obstacle using the ACO, with the parameters given in Equations (1)–(6), as shown in Figure 2 [26].

(1) $$Max\;F\left(y\right)=\varphi_{1}\cdot PC\left(y\right)+\varphi_{2}\cdot PDR\left(y\right)+\varphi_{3}\cdot \;\frac{D_{th}-D\left(y\right)}{D_{th}}\cdot \frac{1}{\left(1+D_{v}\left(y\right)\right)}$$

(2) $$PC\left(y\right)=\overset{n}{\underset{i=1}{\prod }}PC\left(c_{i}\right)\;$$

(3) $$PDR\left(y\right)=\overset{n}{\underset{i=1}{\prod }}PDR\left(c_{i}\right)$$

(4) $$D\left(y\right)=\overset{n}{\underset{i=1}{\sum }}D\left(c_{i}\right)$$

(5) $$D\left(y\right)=\overset{n}{\underset{i=1}{\sum }}\frac{D_{v}\left(c_{i}\right)}{n}$$

(6) $$D\left(y\right)\;\leq \;D_{th}$$

where:

$F\left(y\right)$: Global QoS (from ST to DT).

$Pc\left(y\right),PC\left(ci\right)$: Connection probability.

$PDR\left(y\right),\;PDR\left(ci\right)$: Packet delivery ratio.

$D\left(y\right),D\left(ci\right)$: Delay.

$Dv\left(y\right),\;Dv\left(ci\right)$: Latency variance.

Weight values are φ1, 2, and 3, therefore φ1 + φ2 + φ3 = 1.

### 2.4. Firefighting UGV Terminal Transaction

This section demonstrates the source and destination terminals (ST|DT) technique to link the other pairs. The movement direction of a terminal (source or destination firefighting UGV) and the distance between itself and its nearby junctions, determines a terminal intersection. According to these factors, each candidate junction is assigned a throughput as a cost, and the route with the greatest throughput is chosen as the optimal terminal intersection. Equations (7)–(8) demonstrate the throughput expression [27].

(7) $$Throughput\left(I_{i}\right)=x\cdot \left(1-\frac{d\left(I_{i}\right)}{L}\right)+\left(1-x\right)\cdot Dir\left(I_{i}\right)$$

(8) $$Dir\left(I_{i}\right)=\left\{\begin{array}{c} 1,the\;terminal\;FirefightingUGV\;moves\;to\;I\_i\\ 0,\;otherwise\; \end{array}\right.$$

where:

L: Current length of road segment.

$D\left(li\right)$: Distance between terminal and nearby junction.

Li: Weight parameter.

$Dir\left(li\right)$: Direction value of the communication terminal.

### 2.5. Route Optimization Establishment

In this part, we describe the method of determining the best path from ST to DT. It should be noted that we consider the optimum route problem to be the optimization problem given in Equation (1). To address this obstacle, we provide an ACO-based technique capable of dealing with such nonlinear combinatorial optimization challenges in dynamic and complex systems. Algorithm 1 illustrates the firefighting UAV routing establishment.
**Algorithm 1:** Firefighting UGV Routing Algorithm Establishment1: Update time interval. 2: Determine ST and DT. 3: “S” send a request message to “ST” of routing table towards DT. 4: if there is a high throughput && CurrentTime TGP && D (y) D_th_ then LastUpdateTime. 5: S receives a nice response. 6: else 7: For each arrive packet of UGV at I, do routine If type(A) == forwarding UGV then read Ii’s throughput next intersection next iteration If next iteration == DT then If (D(y) <= D_th_) then Type (A) = backword UGV else Pull (A) End End             End If type (A) == backward UGV update throughput send A to the next intersection If next instruction == ST then update throughput Pull (A) End End                          the best route depends on throughput ST sending a positive routing signal to S 9: “S” starts transmitting packets per intersection. 10> 10: If TIS|TID change. 11: ST employs establish the optimal route. 12: End

#### 2.5.1. Firefighting UGV Route Derivation

The source UGV transmits a routing request to the ST after picking the intersection information for the source and destination. In the case of the DT information, it is available at the ST and is not out of date, the ST responds to S with a positive message; otherwise, the ST responds with non-active message then searches for the best tracking path. To determine the potential tracking route from the ST to DT, the ST sends a set of forwarding signals through the road’s fire sensor, with the number of forwarding signals set to the UGV. Whenever a forward signal arrives at the fire sensor intersection Ii, it saves the transmission throughput before making a random choice to select, depending on the throughput, the next intersection recorded at Ii, assuming that Ii has K surrounding crossings, namely I1, I2,..., IK1, IK. The probability pij with the firefighting UGV picks the next “Ij”, which is illustrated in Equations (9)–(11). When a forward signal from a firefighting UGV arrives at the DT with a delay of less than the Dth, the firefighting UGV’s path is selected as an available candidate route [28].

(9) $$P_{ij}=\frac{LP_{ij}^{\propto }\;\cdot GP_{ij}^{\beta }}{\sum_{m=1}^{k}LP_{im}^{\propto }\cdot GP_{im}^{\beta }}$$

(10) $$LP_{ij}=F\left(c_{ij}\right)=\varphi_{1}\cdot PC\left(c_{ij}\right)+\varphi_{2}\cdot PRD\left(c_{ij}\right)+\varphi_{3}\cdot \frac{D_{th}-D\left(c_{ij}\right)}{D_{th}}\cdot \frac{1}{\left(1+D_{v}\left(c_{ij}\right)\right)}\;$$

(11) $$GP_{ij}=F\left(y_{ij}\right)=\varphi_{1}\cdot PC\left(y_{ij}\right)+\varphi_{2}\cdot PRD\left(y_{ij}\right)+\varphi_{3}\cdot \frac{D_{th}-D\left(y_{ij}\right)}{D_{th}}\cdot \frac{1}{\left(1+D_{v}\left(y_{ij}\right)\right)}$$

where:

α and β: represent the weight values

LPij: represents the local throughput, which indicates the quality of service e_ij_ (Ii-I_j_).

GPij: is denoted as the general throughput.

$PC\left(cij\right)$: connection probability, $PDR\left(cij\right)$: packet delivery ratio, $D\left(cij\right)$: delay, and $Dv\left(eij\right)$: delay variance of road segment.

The same QoS measures for route yij are represented by $PC\left(yij\right)$, $PDR\left(yij\right)$, $D\left(yij\right)$, and $Dv\left(yij\right)$. LPij, which is a heuristic throughput that can assist forward the UGV in obtaining the most recent local QoS information and exploring the best routing pathways.

GPij: indicates the optimal QoS to the UGV, benefiting the algorithm convergence and avoiding routing difficulties on the road.

*p_ij_*: is used to maintain the stability of both the new route exploration and the existing route utilization.

#### 2.5.2. Route Optimization

In terms of the updated global throughput, we use the backward UGVs to conduct the optimal route selection among the candidate routes considered. When a forward UGV signal arrives at the DT, it is turned into a backward UGV if the delay of transmission meets the requisite Dth (threshold delay); otherwise, it is discarded straight. This feedback UGV transports an intersection list before returning to the ST through the same but reverse path, as the comparable forward UGV. When the backward UGV arrives at a specific Ii, it first calculates the latest global throughput *LGT_ij_* using the transported throughput *GT_ij_* [29,30].

(12) $$GT_{ij}\leftarrow \left(1-\sigma \right)\cdot GT_{ij}+\sigma \;LGT_{ij}\;$$

(13) $$GT_{ij}\left(i+T_{eva}\right)\;\left\{\begin{array}{c} \eta \cdot GT_{ij}\left(t\right)\;if\;GT_{ij}\left(t\right)>\tau_{min}\\ \tau_{min}\;Otherwise \end{array}\right.$$

where:

*σ*: is the weighting parameter (0, 1). Obviously, this throughput updating procedure may prevent the routing path exploration stagnation and mitigate the influence of the *LGT_ij_*’s immediate value. Following that, $GT_{ij}$ is refreshed in the field of this backward ant’s updated global throughput.

Once all reverse UGVs are at TIS, the $F\left(y\right)$ values of all possible candidate routes are compared, and the route with the highest $F\left(y\right)$ value is chosen as the best. The ST then sends a positive routing message to the source vehicle to begin the data packet forwarding.

## 3. Result and Discussion

We analyze the suggested public road fragment QoS before comparing it to several versions of the global routing protocols (CAR [10], GSR [20]) and assisted routing protocols (EIGRP [21], SADV [12]). To further understand the benefits of the suggested model, we define the VANET-ACO as a modified VANET, in which the tracking route choices are studied for the terminal-per-terminal vehicles network rather than the ST-per-DT.

### 3.1. The Experimental Setting

In these simulation studies, we use the VANET simulator (firefighting UGV), as indicated in Table 2. The simulation area is 5000 m × 5000 m, with 57 crossroads and 91 road segments, and the cars drive at speeds ranging from 10 to 20 m/s. The vehicle spacing density is 0.01-0.04 automobiles per meter, and the vehicle safety time is 2 s (s). As the mobility model, the intelligent unnamed ground vehicles model with intersection management is employed, and the vehicles approaching the junctions are set to obey the strategy that says “first arrive, first go through, and turn right”. Furthermore, the starting points also the tracking routes of the automobiles set at random; Omnt++ has been utilized as the simulator to produce 250 throughput (TH) sessions stochastically. To reduce the random effects for the UGV, and we perform each simulation 50 times to obtain the adequate confidence intervals (CI).

### 3.2. QoS Validation and Analysis

**Figure 3:**
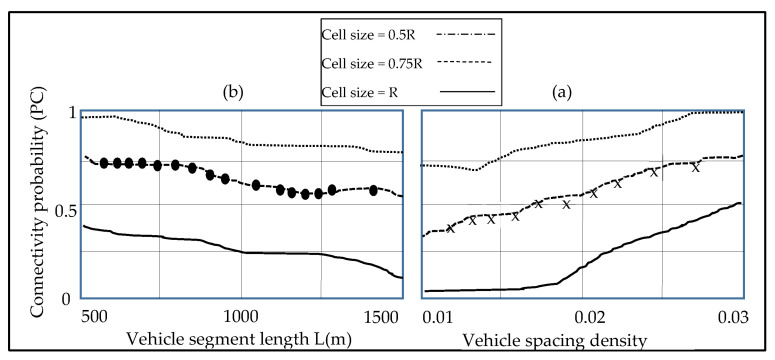
Road signal connectivity depends on the connectivity and density. (**a**) shows the upper and lower bounds of the road segment connectivity for cs = R and cs = 0.5 R. Accordingly, the average analytical results (CI = 95%) closely match the simulation results for cs = 0.75 R. We set L = 1500 m and cs = 0.75 R in (**b**).

**Figure 4:**
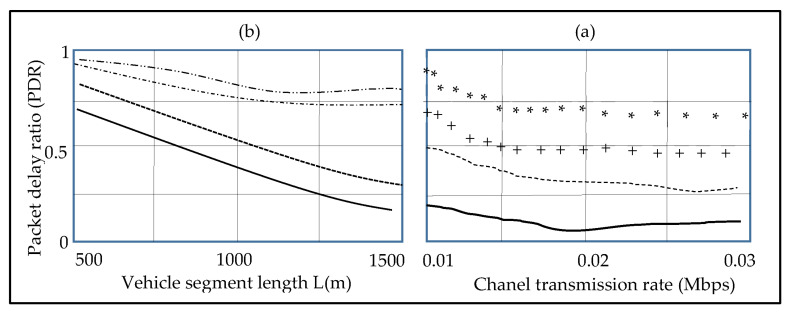
Road segment packet delivery ratio, depends on the channel transmission rate. (**a**) the mean distance between the theoretical and simulation results is only 4.85%, in the case of cs = 0.75 R. Furthermore, as the distance of the road segment increases, so does the ratio of the packets delivered, since more transmission hops are necessary. (**b**) depicts the packet delivery ratio model’s availability with the varied vehicle separation density, packet size, and the channel transmission rate (CTR).

Figure 3a,b show how the road segment length L, cell size cs, vehicle distance density, and communication range R affect the road segment probability. Figure 3a shows the upper and lower bounds of the road segment connectivity for cs = R and cs = 0.5 R. Accordingly, the average analytical results (CI = 95%) closely match the simulation results for cs = 0.75 R. We set L = 1500 m and cs = 0.75 R in Figure 3b, and we notice that the mean analytical findings match the simulation model for all Rs. The packet delivery ratio of the road segment is adjusted at λ = 0.015 vehicles/m in Figure 4a, the mean distance between the theoretical and simulation results is only 4.85%, in the case of cs = 0.75 R. Furthermore, as the distance of the road segment increases, so does the ratio of the packets delivered, since more transmission hops are necessary. Moreover, when the interference percentage is reduced due to less interference from the surrounding nodes, the packet delivery rate increases.

**Figure 5:**
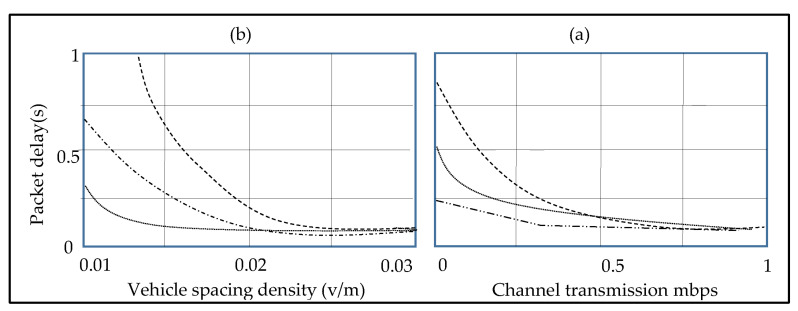
Road segment packet delay in the transmission channel. (**a**,**b**) depict the relationship between the road section delay, cell size, CTR, and the spacing density of the UGV.

Figure 4b depicts the packet delivery ratio model’s availability with the varied vehicle separation density, packet size, and the channel transmission rate (CTR). As anticipated, as the CTR increases, the delivery ratio of the packet decreases, resulting in an increased BER, and a lower SINR. Furthermore, as the vehicle distance density grows, the ratio of the packet delivery increases also. This is due to the fact that the increasing UGV spacing density enhances the network connectivity, forcing the data packets to be routed mostly over wireless communications, which causes more interference from the wireless channel decaying. Figure 5a,b depict the relationship between the road section delay, cell size, CTR, and the spacing density of the UGV.

Once the simulation and the analytical results concur well for cs = 0.75 R, the precision of our road segment delay model is demonstrated. Additionally, from Figure 5a it shows that, when the vehicle spacing density grows, the road segment latency decreases significantly, demonstrating that the network partitions may be corrected. In Figure 5b, the “λ” is set to (0.03) vehicles/m. The figure illustrates how the latency of the road segments reduces as the CTR improves, allowing the packets to communicate swiftly with no need for consuming time.

Based on the previous study, in the case of cs = 0.75 R, the whole error average for the simulation and the analytical findings are not more than 5%, demonstrating that, the suggested QoS scheme of the road segments are viable. Furthermore, in the case of the vehicle density increases, the connection possibility increases, the latency falls, although delivering the ratio of the packets will fall. The findings of the study suggest three QoS metrics to be combined, in order to attain a complete QoS routing performance.

## 4. Conclusions

A novel protocol to enhance the VANETs using the swarm technique for adaptively picking the appropriate routing path for firefighting UGVs, is proposed in this paper. Initially, the route was viewed as the optimization problem, that could be solved by inventing the ACO method. Using the throughput of a wireless node on each firefighting UGV forwards, the firefighting UGVs investigate the potential candidate routing options between the terminal crossings, and then the corresponding backward firefighting UGVs determine the ideal route and update the latest throughput. Furthermore, we developed QoS models in the road segment situations, by taking three metrics into account, the connection chance, packet delivery ratio, and the latency. Finally, by using extensive simulations, we verified our analytical QoS models and proved that the new enhanced VANETs outperform the GSR and CAR reference paradigms. In the next study, We would like to look at the impacts of the VANET weight factors on the road segment relaying quality, as well as comparing the ACO’s performance to other optimization methods.

## Figures and Tables

**Table 1:** The prior studies along with their limitations.

Reference	Limitation
[6]	Some research has employed VADD and A-STAR to address the problem of the QoS, but it remains a theory that is not appropriate for dealing with dynamic networks, such as VANETs.
[7]	Other research employed SADV to redirect packets and increase the QoS in real-time. However, this strategy might cause a network congestion, especially during peak hours.
[4,5]	Some researchers have employed local motion characteristics, such as the GyTAR and TADS protocols to predict the next junction, although neither the density of the chemicals nor their distribution can reflect the overall pattern of the QoS improvement. The increased vehicle density can improve the network connectivity, but it also can cause an increase in data throughput on the network.
[8]	The CAR protocol for the routing packets employs the idea of blind flooding in its schemes, rendering it ineffectual in improving the QoS and potentially burdening the network.

The references represent the main targeted references for the study.

**Table 2:** Shows the simulation settings in the proposed study.

Parameters	Values
The network protocol	IEEE 802.11p
The packets rate	2 pps
The range of communication	150–300 m
Transmission range	250–400 m
Packet size	64 B
Number of firefighting UGVs	50–3000
Delay	10 ms
α, β parameter	0.5, 0.3
σ parameter	0.2
QoS weights φ1,2,3	0.2, 0.2, 0.6
Speed of vehicles	10 to 20 m/s
Time simulation	30 min
Simulation range	[5000 × 5000] (m)

α, β, σ parameter chosen values after several itterations.

## Data Availability

Not applicable.
